# Abstracts

**Published:** 1894-08

**Authors:** A. H. Goelet


AN OPERATION FOR THE RADICAL CURE OF FISTULA IN ANO BY AN IMPROVED METHOD WHICH SECURES PRIMARY UNION AND COMPLETE RETENTIVE POWER, EVEN WHEN TWO INCISIONS THROUGH THE SPHINCTER ARE NECESSARY.—In an article which appeared in the July number of the *American Medico-Surgical Bulletin* under the above title, Dr. A. H. Goelet, of New York, described an operation which, in his hands, has been very successful. As in the older methods, the sphincter is completely divulsed, and the fistula opened into the rectum and thoroughly curetted. The important part of the operation, however, lies in the method of suturing. This is as follows: In the deeper structures, two or more rows of buried, continuous sutures of fine catgut are employed, each row beginning at the upper angle beneath the mucous membrane, and ending just within the integument of the perineum covering up the preceding row. In the rectal mucous membrane and the integument of the perineum, interrupted sutures of chromic catgut are used. The edges of the sphincter muscles are approximated with especial care.

Deep sutures introduced through the rectal mucous membrane are deprecated by the operator, because of the danger of leakage of septic matter along the track of the sutures, and because they obstruct the circulation and increase the œdema, thus interfering with primary union.

Dr. Goelet reported a case where two fistulæ existed, which had been treated after this method. The result was very gratifying. The wound healed by primary union, and although the external sphincter had been cut in two places, at opposite points, and the internal sphincter at one point, the patient had complete retentive power. This result contrasted with that obtained by the older methods, establishes this as an ideal operation.

The article concluded by emphasizing these essential details in the technique of the operation:

1. Complete divulsion of the sphincter.
2. Perfect asepsis.
3. Incision of the muscles at right angles to the fibres.
4. Thorough curettage of all fistulous tracts.
5. The use of buried sutures of fine catgut for the deeper structures, and interrupted chromic catgut sutures for the mucous membrane.
6. The rectal tube and dressing.
7. Absolute inactivity of the bowels and a liquid diet for five days following the operation.

---

LIGATION OF THE BASE OF THE BROAD LIGAMENTS PER VAGINAM, INCLUDING THE UTERINE ARTERIES, FOR FIBROIDS OF THE UTERUS.—Dr. Augustin H. Goelet, of New York, in a contribution to the *American Medico-Surgical Bulletin*, June 1st, reports favorably upon this operation, in his hands, for the control of uterine hemorrhage and reduction of fibroid growths. He believes it should be done in lieu of hysterectomy, when that operation would involve too great a risk, and as a preliminary step with a view of avoiding the necessity of the more hazardous operation. When extensive attachments have not been formed which would afford additional nutrition, considerable reduction has resulted even in growths of large size. When the operation has been done for smaller growths, the result has been more satisfactory. In some instances, complete atrophy has been reported. This result, as well as arrest of the uterine hemorrhage, is accounted for by the diminished nutrition furnished the uterus and these growths by the interference with the blood supply and nerve supply, which are included by ligation of the base of the broad ligaments. It is estimated that the uterine arteries furnish the uterus with two-thirds of its blood supply, and it is reasonable to expect that a profound effect will be produced upon that organ, and growths arising from the walls, if this is suddenly cut off.

The sole danger in the operation is the risk of including the ureters in the ligatures, as they pass down behind the uterine arteries only half an inch from the cervix, and are consequently in the field of operation. Dr. Goelet suggests as a preliminary step, to eliminate this risk, that bougies be passed into the ureters through the bladder. He admits, however, that a careful operator, accustomed to working in this region, may easily avoid the ureters.

The technique of the operation, as described by Dr. Goelet, shows an important departure from the usual method followed. Instead of ligating each artery in only one place on a level with the internal os, he applies a second, and often a third ligature to the artery on each side as it ascends along the side of the uterus, the result of which is to cut off the compensating blood supply from ovarian artery to the lower part of the uterus.

Dr. Goelet gives all the credit of priority to Dr. Martin, of Chicago, who has recently suggested and popularized the operation and perfected its technique, but states that he first ligated the uterine artery *per vaginam* on one side in January, 1889, in the case of a large fibroid the size of a seven months' pregnancy, with a view of diminishing the size of the growth by reducing the blood supply. The artery on the other side was not ligated, because the position of the tumor made it inaccessible. Six months later the tumor was one-third smaller, and was giving no inconvenience.

He quoted his last case operated upon, to show how promptly uterine hemorrhage may be controlled by this operation.

